# Smart greening initiatives for healthcare facilities: A caribbean case study on environmental sustainability and disaster resilience

**DOI:** 10.1016/j.joclim.2025.100525

**Published:** 2025-10-09

**Authors:** Judith Harvey, Dana van Alphen, Shalini Jagnarine Azan, Roger Camacho, Clemens Buter, Ciro Ugarte, Lealou Reballos, Alex Camacho-Vasconez, Juan S. Izquierdo-Condoy, Jorge Vasconez-Gonzalez, Esteban Ortiz-Prado

**Affiliations:** a PAHO/WHO International Consultant, United States; b Health Emergencies Department, PAHO/WHO, Washington D.C, United States; c One Health Research Group, Universidad de las Americas, Quito, Ecuador

**Keywords:** Smart hospitals, Green healthcare, smart hospitals, Environmental sustainability, Disaster Resilience, Energy Efficiency

## Abstract

**Background:**

The Smart Hospitals Program was implemented in seven Caribbean countries to enhance disaster resilience and environmental sustainability in small- to medium-sized healthcare facilities. The initiative focused on improving energy efficiency, water conservation, and overall environmental performance while ensuring uninterrupted healthcare service delivery in disaster-prone regions.

**Methods:**

Facility assessments were conducted using the Green Checklist, a tool tailored for Caribbean healthcare facilities based on LEED standards. Pre- and post-retrofit evaluations targeted water and energy conservation, air quality, and waste management. Interventions included infrastructure upgrades—such as photovoltaic systems, energy-efficient equipment, and rainwater harvesting systems—and staff training on resource conservation.

**Results:**

Energy consumption at the Vieux Fort Healthcare Facility decreased by 47.7 %, with a mean monthly reduction of -5773 kWh (95 % CI:6175 to -5371; *p* < 0.0001; *t* = 31.61, df = 11). Comfort Bay showed a median reduction of 26.6 % (-1919 kWh; *p* = 0.0005; *W* = -78.00). Water consumption reductions included 58.5 % at Saltibus (-40,456 gallons per month; *p* = 0.0005; *W* = -78.00), 18.1 % at Vieux Fort (-6924 gallons per month; *p* = 0.0005; *W* = -78.00), and 7.2 % at Mongouge (-1269 gallons per month; *p* = 0.0005; *W* = -78.00). These reductions highlight significant improvements in resource efficiency and operational sustainability across facilities.

**Conclusions:**

The Smart Hospitals Program achieved significant reductions in water and energy consumption, demonstrating the potential of targeted retrofitting interventions to enhance sustainability and resilience in healthcare facilities. While the results highlight the program's effectiveness, maintenance challenges and variability in usage patterns underscore the need for ongoing monitoring and tailored strategies to sustain these gains. This model provides valuable insights for similar initiatives in resource-constrained and disaster-prone regions.

## Graphical abstract

**Figure:**
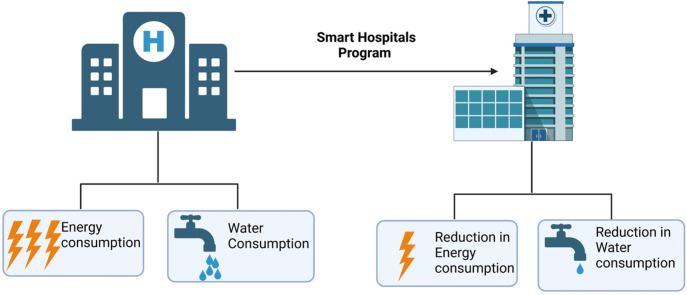

## 1 Introduction

Energy consumption has increasingly become a global priority, not only due to rising costs but also because even clean energy sources, such as thermoelectric and hydroelectric power, are facing challenges caused by climate change, which has already impacted various regions worldwide [1,2]. Energy is essential for modern civilization, and critical infrastructure such as healthcare facilities—particularly hospitals—requires a reliable energy supply [3,4]. However, these facilities are among the most energy-intensive structures within societal systems [3,4]. Their continuous operation, advanced medical equipment, and high demand for heating, cooling, and water contribute to a significant environmental footprint [5]. The global healthcare sector significantly impacts the environment, contributing approximately 5.2 % of global emissions, equating to 2.7 Gt CO2 equivalent, as reported in the 2022 Lancet Countdown. In some developed countries, such as the Netherlands, this figure rises to 7 %, emphasizing the critical need for targeted sustainability initiatives within the sector [6]. These institutions, while critical for public health, face growing challenges from climate change, which threatens their operational stability and increases their environmental responsibility [7].

Hospitals, while vital for public health, disaster risk reduction, emergency preparedness, response, and recovery, face escalating challenges from climate change, which jeopardizes their operational stability and amplifies their environmental impact [8]. In emergency situations, countries need functional healthcare facilities to treat the sick and injured despite the disaster unfolding around them [9]. Consequently, enhancing energy efficiency, minimizing carbon emissions, and integrating sustainable practices have become essential pillars of modern healthcare infrastructure. While climate change affects all regions of the planet, vulnerable zones like Latin America and the Caribbean are particularly at risk due to factors such as population density and inequity [[10], [11], [12]]. The Caribbean, in particular, faces unique challenges as many of its small islands often lack the capacity to generate sufficient electricity from sources like thermal or hydropower, making energy sustainability a critical concern [13]. Additionally, the region is highly exposed to natural hazards such as hurricanes, tropical storms, floods, and droughts, which not only threaten healthcare infrastructure but also disrupt essential health services during emergencies [11,[14], [15], [16]]. Moreover, as a region heavily reliant on tourism, the resilience and sustainability of its healthcare systems have broader implications for economic stability and emergency preparedness [17].

In this context, we present the findings of the Smart Hospitals Program, implemented across seven Caribbean countries between 2015 and 2023. This program's locally gathered data offers valuable insights into the effectiveness of targeted interventions in enhancing sustainability and resilience within healthcare facilities, providing a practical model for addressing similar challenges in other regions.

## 2 Materials and Methods

### 2.1 Study Design and Setting

This pre-post intervention study was conducted across seven Caribbean countries between 2015 and 2023 as part of the Smart Hospitals Program. The program targeted small- to medium-sized healthcare facilities, including homes for the elderly, to implement retrofitting measures to enhance safety and environmental sustainability. The facilities were geographically diverse, with varying access to water and energy resources, contributing to the generalizability of the findings

### 2.2 Participants and Facilities

The study focused on four healthcare facilities in Saint Lucia, strategically selected based on their size, geographical location, and operational capacity to ensure a representative understanding of regional healthcare challenges. These facilities included small healthcare centers serving fewer than 50 patients daily and homes for the elderly, which are critical to the region's healthcare framework. The selection of these sites allowed for a comprehensive evaluation of retrofitting interventions across diverse healthcare settings.

### 2.3 Data Collection

Data were collected pre- and post-retrofitting to evaluate the impact of interventions on water and energy usage. Quantitative data included monthly water and electricity consumption records. These measurements were independently verified against utility company reports for accuracy and consistency. Qualitative aspects, such as indoor environmental quality and waste management practices, were also assessed but are not discussed in detail in this paper.

### 2.4 Green Checklist and Assessment Criteria

The Green Checklist incorporated scoring calculations with weighted questions, prioritizing critical areas such as water and electricity conservation. Key assessment categories included indoor environmental quality, atmospheric management, hazardous materials handling, pharmaceutical and food services, and solid and infectious waste management. Quantitative data collection captured water and electricity consumption at each facility before and after retrofitting. Specific interventions included transitioning to energy-efficient lighting and air conditioning systems and implementing rainwater harvesting measures to mitigate environmental impacts.

### 2.5 Facility Interventions and Procedures

Pre- and post-retrofit assessments were conducted using the Green Checklist, a tool specifically tailored for the Caribbean region and modeled on Leadership in Energy and Environmental Design (LEED) standards. This checklist evaluated sustainability and safety across facilities, assigning percentage scores up to a maximum of 100 %. In collaboration with stakeholders, a "Gold Standard" threshold of 70 % or higher was established to signify optimal greening efforts. The checklist included weighted scoring and prioritizing key water and electricity conservation areas.

The retrofitting process included infrastructure modifications and procedural enhancements, essential to improving facility sustainability. Infrastructure upgrades comprised the installation of low-flow plumbing fixtures, energy-efficient Light Emitting Diodes (LED) lighting systems, inverter air-conditioning units, and photovoltaic systems (where feasible) to reduce reliance on grid electricity. Targeted staff training sessions complemented these changes and focused on energy and water conservation practices. Training topics included efficient appliance use, monitoring resource consumption, and prompt reporting of maintenance issues. To support the long-term sustainability of these interventions, facility managers were provided with detailed handover packages, including as-built drawings of the new water and electrical systems.

The following table (Table 1) summarizes the key interventions and their objectives across different categories:

**Table 1:** Summary of Key Interventions and Objectives in Retrofitting Healthcare Facilities. This table outlines the categories of retrofitting interventions implemented in healthcare facilities, their specific measures, and the objectives they aim to achieve. The interventions targeted water conservation, energy efficiency, staff training, and predictive modeling to optimize sustainability and resource management.

Category	Intervention/Measure	Objective/Focus
Water Conservation	Low-flow plumbing fixtures	Minimize water consumption
	Leak repairs	Prevent water wastage
	Increased storage capacity	Ensure water availability during shortages or emergencies
	Rainwater harvesting systems	Collect and utilize rainwater for non-potable applications or broader facility use
	Proposed future improvements: gray-water reuse and wastewater reclamation	Reduce dependency on treated mains water and ensure sustainable water management
Energy Efficiency	Energy-efficient upgrades (e.g., LED lighting systems, inverter air-conditioning units)	Minimize energy consumption
	Renewable energy systems (e.g., photovoltaic solar systems)	Supplement energy needs and reduce grid dependency
	Sealed air-conditioned spaces	Minimize energy loss and improve cooling efficiency
	Occupancy sensors	Reduce unnecessary energy consumption by turning off appliances when spaces are unoccupied
Staff Training	Conservation practices (e.g., appliance usage, maintenance reporting, monitoring energy/water usage)	Support sustainable operational practices
Predictive Models and Estimates	Water consumption (faucets, water closets, washers, sterilizers)	Identify key areas for conservation
	Electricity consumption (lighting, cooling systems)	Predict energy savings from retrofitting
	Conversion to carbon dioxide equivalents (gCO2e)	Evaluate the environmental impact of reduced energy consumption
	Rainwater harvesting systems	Include in planning but savings not yet quantified

### 2.6 Energy Audits and Renewable Energy

Specialized energy audits were conducted before the retrofitting process to establish baseline energy consumption and identify areas for improvement. These audits assessed the performance and efficiency of energy-intensive systems, such as cooling systems, lighting, and other electrical appliances. The audits also estimated potential energy savings achievable by adopting advanced technologies, including LED lighting and inverter air-conditioning units. Where feasible, renewable energy sources, such as solar and wind power, were evaluated and incorporated into facility operations to reduce reliance on grid electricity and minimize environmental impact. These renewable energy solutions were tailored to each facility's specific needs and geographic conditions, further enhancing the program's sustainability goals.

### 2.7 Consumption Estimates and Predictive Models

The Baseline Assessment Tool (BAT) was employed before retrofitting to estimate potential water and energy consumption savings across facilities. The BAT identified energy-intensive equipment and correlated its usage with the number of occupants and the range of services provided. The focus was on faucets, water closets, washers, and sterilizers for water efficiency. For electricity, key areas include lighting and cooling systems. Savings were estimated based on transitioning to water- and energy-efficient alternatives. Rainwater harvesting systems were proposed for non-potable purposes, though their savings were not quantified, and provisions were made for potential system failures during droughts.

Electricity savings were calculated in kilowatt-hours (kWh) and converted into carbon dioxide equivalents (gCO2e) to evaluate environmental impact. For instance, replacing fluorescent and incandescent lighting with LED fixtures and upgrading air-conditioning units to inverter models yielded significant reductions in energy consumption and greenhouse gas emissions. Table 2 provides an example of estimated savings for two healthcare facilities in Saint Lucia. These estimates assumed no change in the total number of lights or the volume of air-cooled.

**Table 2:** Energy Savings Estimates. This table summarizes the annual electricity savings, carbon dioxide reductions, and monetary savings achieved through lighting and air-conditioning upgrades in two healthcare facilities in Saint Lucia. Calculations are based on a rate of $0.32 per kWh (November 2022) and a carbon emission factor of 655 gCO2e per kWh.

Facility Name	Annual Saving (kWh)	Annual Carbon Saving (kgCO2e)	Monetary Saving (USD)
Comfort Bay Senior Citizens’ Home	17,763	11,635	$5684.16
Vieux Fort Wellness Centre	25,318	16,583	$8101.76

The greenhouse gases in older air-conditioning units were phased out as part of the retrofitting process. Although this phase-out could result in additional CO2e savings, it was not estimated due to uncoordinated deconstruction processes across participating countries. The contribution of photovoltaic (PV) systems to energy savings was also not included in the calculations; however, many PV systems were grid-tied, and their renewable energy contributions were likely reflected in national carbon-equivalent factors for energy generation.

To predict kWh production from photovoltaic installations, estimates considered peak sunlight hours, weather patterns, cloud cover, panel efficiency, and orientation. Typically derived from manufacturer software, these projections were accessible for several project installations.

### 2.8 Cost-Benefit Analysis

The Retrofitting Economic Support Tool (REST), developed by the Department of Health Policy and Management at Florida International University and integrated into the Smart Hospitals Toolkit by the Pan American Health Organization (PAHO), was employed to assess the cost-effectiveness of the retrofitting projects. REST provided a comprehensive analysis of both economic and health-related outcomes by incorporating pre- and post-retrofit data on safety and sustainability improvements.

Key inputs for the tool included energy and water consumption data, projected retrofitting costs, and quality-adjusted life years (QALYs) gained due to improved facility resilience and efficiency. REST generated a detailed cost-effectiveness analysis (CEA), presenting metrics such as net costs, return on investment (ROI), and incremental cost-effectiveness ratios (ICER). Facilities with negative net costs (indicating savings exceeded expenditures) or high ROI (greater than $1 return for every $1 invested) were prioritized.

The tool also calculated QALYs to evaluate the health benefits of retrofits, with considerations for both disaster preparedness and operational sustainability. REST outputs, such as graphs and tables summarizing cost and benefit metrics, guided the decision-making process to identify facilities that offered the most substantial economic and health returns.

By comparing actual post-retrofit data with predicted savings, REST validated the economic feasibility of the interventions and identified opportunities for further optimization. This ensured that investments in healthcare infrastructure yielded tangible benefits in cost savings and enhanced resilience.

### 2.9 Water Conservation Planning and Efficiency

Water conservation initiatives targeted critical areas to optimize resource use and ensure sustainable operations. Key measures included installing low-flow plumbing fixtures to minimize water consumption, prompting leak repairs to prevent wastage, and expanding storage capacity to enhance water availability during shortages or emergencies. Rainwater harvesting systems were implemented to collect and utilize rainwater for non-potable applications or treat it for broader facility use.

In addition to these interventions, the Green Checklist provided recommendations for future improvements, such as integrating gray-water reuse systems and adopting wastewater reclamation technologies. These enhancements aim to reduce water dependency further and promote long-term sustainability within healthcare facilities.

### 2.10 Statistical Analysis

Statistical analysis was conducted to evaluate the impact of retrofitting interventions on water and electricity consumption across the facilities. Water consumption (measured in liters per day per facility) and electricity consumption (measured in kilowatt-hours per day) were analyzed using paired *t*-tests to determine the statistical significance of changes between pre- and post-retrofitting values. Key metrics were calculated to quantify the reductions observed, including mean differences, standard deviations, and 95 % confidence intervals. The analysis was performed using SPSS software, version 24, ensuring rigorous statistical evaluation and reliability of results. Correlation coefficients were calculated to assess the effectiveness of pairing in detecting intervention impacts. Graphical representations of the results were created using Prism 10 software for clarity and visualization. All statistical tests were two-tailed, with a significance level set at *p* < 0.05.

## 3 Results

### 3.1 Energy consumption measurements after retrofitting

**Fig. 1:**
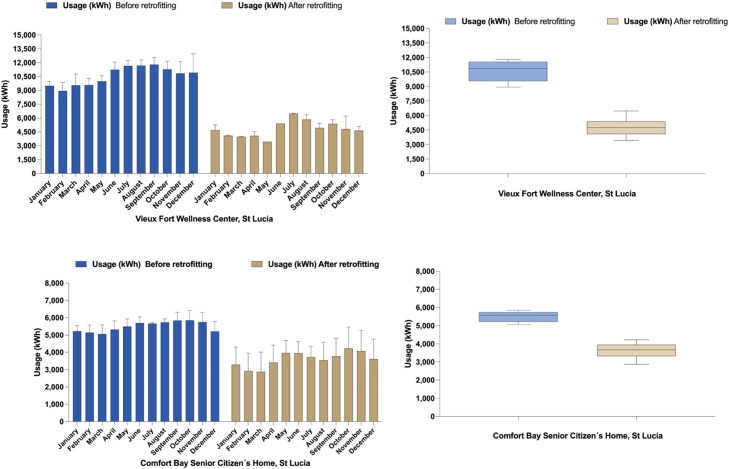
Monthly and annual electricity consumption before and after retrofitting at two healthcare facilities in Saint Lucia.

Energy consumption analysis for Vieux Fort and Confort Bay Healthcare Facilities demonstrated significant reductions following retrofitting interventions. For Vieux Fort, the mean monthly decrease was −5773 kWh (95 % CI: −6175 to −5371; *p* < 0.0001; *t* = 31.61, df = 11), with consistent savings averaging 5.77 MWh. Variability in differences was minimal (SD = 632.7 kWh, SEM = 182.6 kWh), and an R-squared value of 0.9891 indicated that 98.91 % of the variability in energy savings was linked to the interventions. Pre-retrofitting monthly energy usage ranged from 7896 kWh to 13,070 kWh, decreasing to 3428 kWh to 6520 kWh after retrofitting, with notable reductions in high-demand months such as May (4468 kWh) and December (7612 kWh) (Fig. 1).

For Confort Bay, the median monthly decrease in energy usage was −1919 kWh (*p* = 0.0005; Wilcoxon signed-rank test, *W* = −78.00), with all differences reflecting reductions in consumption. The Spearman correlation coefficient (rs = 0.8112, *p* = 0.0011) indicated a strong relationship between pre- and post-retrofitting data. Monthly energy usage before retrofitting ranged from 7500 kWh to 12,500 kWh, with significant reductions observed across all months after retrofitting.

### 3.2 Water consumption measurements after retrofitting

Water consumption data from three healthcare facilities in Saint Lucia—Saltibus Healthcare Facility, Mongouge Health Center, and Vieux Fort Healthcare Facility—demonstrated significant reductions following retrofitting interventions. At the Saltibus Healthcare Facility, water usage decreased by a median of −40,456 gallons per month (*p* = 0.0005; Wilcoxon matched-pairs signed rank test, *W* = −78.00), with strong pairing effectiveness confirmed by a Spearman correlation coefficient of 0.7552 (*p* = 0.0031), indicating a substantial relationship between pre- and post-retrofitting data.

At the Mongouge Health Center, water consumption showed a statistically significant median reduction of −1269 gallons per month (*p* = 0.0005; *W* = −78.00). However, pairing effectiveness was not significant (Spearman correlation coefficient = 0.1049, *p* = 0.3746), suggesting limited correlation between pre- and post-retrofitting data, potentially due to variability in usage patterns.

**Fig. 2:**
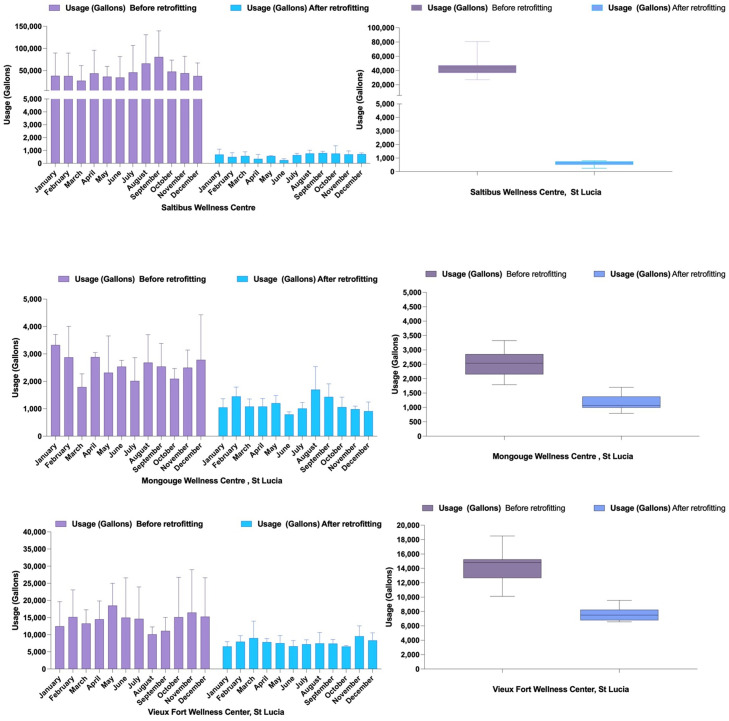
Monthly and annual water usage before and after retrofitting at three healthcare facilities in Saint Lucia.

Similarly, the Vieux Fort Healthcare Facility achieved a significant median monthly reduction of −6924 gallons (*p* = 0.0005; *W* = −78.00), but the pairing effectiveness was weak (Spearman correlation coefficient = 0.3427, *p* = 0.1381). Despite the lower correlation at Mongouge and Vieux Fort, all three facilities recorded meaningful decreases in water consumption, highlighting the overall effectiveness of the retrofitting efforts (Fig. 2).

## 4 Discussion

The findings from the retrofitting interventions in healthcare facilities across Saint Lucia, encompassing Saltibus, Mongouge, Vieux Fort, and Comfort Bay, demonstrate the tangible benefits of integrating water and energy efficiency measures into healthcare infrastructure. The significant reductions in utility consumption across these facilities underscore the efficacy of targeted interventions, combining infrastructure upgrades with staff training to enhance resource efficiency and operational sustainability.

Water conservation efforts yielded notable results, particularly at the Saltibus Healthcare Facility, where significant reductions were achieved through leak repairs and the installation of low-flow plumbing fixtures. These measures addressed visible and underlying infrastructure issues, demonstrating that resolving maintenance challenges is essential for sustainable resource management.

Similarly, Mongouge saw steady declines in water consumption post-retrofit due to water-efficient upgrades, although the smaller operational footprint resulted in more modest overall reductions. At Vieux Fort, the implementation of rainwater harvesting systems for non-potable uses, such as toilet flushing, proved particularly impactful, offsetting increased demand from the addition of washrooms and plumbing fixtures [18,19]. This result highlights the potential of rainwater harvesting systems to mitigate water scarcity in resource-constrained environments.

Energy efficiency interventions also demonstrated marked success. The Vieux Fort facility, for instance, achieved substantial reductions in electricity consumption following the replacement of outdated systems with energy-efficient LED lighting, inverter air-conditioning units, and the installation of a photovoltaic (PV) system. The integration of renewable energy was particularly effective in reducing reliance on grid electricity and supporting high-energy-demand systems like central air conditioning [20,21]. Comfort Bay Home similarly benefited from retrofitting, but savings were less consistent due to external factors such as the addition of perimeter lighting and temporary PV system shutdowns. These fluctuations underscore the need for robust maintenance protocols to ensure sustained efficiency gains and mitigate unforeseen increases in energy demand.

The combined impact of infrastructure upgrades and behavioral interventions, such as staff training, reinforced the sustainability of these efforts. Training sessions focused on practical conservation practices, including identifying leaks, monitoring resource usage, and optimizing appliance settings [22,23]. These behavioral changes complemented the physical retrofits, further enhancing the long-term efficacy of the interventions. However, over time, varying levels of staff engagement highlight the importance of ongoing education and incentivization to maintain adherence to conservation practices.

The results also reveal critical lessons for future retrofitting initiatives. Effective retrofitting requires a comprehensive approach addressing structural inefficiencies and behavioral practices. Integrating rainwater harvesting systems was most successful for water conservation when complemented by plumbing fixture upgrades, while energy efficiency efforts were most impactful when high-consumption areas were targeted. Predictive models for savings could be refined to incorporate facility-specific variables, such as operational demands and local environmental conditions, to improve accuracy and set realistic targets for resource conservation.

Maintenance was also identified as a critical component in preserving the gains achieved through retrofitting. Health facilities should adopt proactive maintenance strategies involving regular inspections and timely repairs to avoid equipment degradation and resource losses. The Green Checklist’s recommendation for periodic reassessment every five years provides a valuable framework for tracking progress and identifying opportunities for further improvement [24]. Moreover, refresher training sessions can reinforce sustainable practices and address emerging challenges. In parallel, the Smart Hospitals Toolkit offers a suite of tools designed to support the resilience of healthcare facilities to climate-related and other disasters [25,26].

## 5 Limitations

Despite these successes, the program faced limitations. Variability in resource availability, such as inconsistent rainfall for rainwater harvesting systems and differing levels of maintenance support across facilities, highlighted the need for adaptable strategies tailored to local conditions. Furthermore, while initial training proved effective, maintaining high levels of staff engagement in conservation efforts requires sustained educational and motivational efforts.

The Smart Hospitals Program’s outcomes underscore the importance of integrating sustainability into healthcare infrastructure, particularly in resource-constrained or disaster-prone regions. Future research should focus on refining cost-benefit analyses to assess the long-term economic impacts of retrofitting and exploring predictive tools that incorporate facility-specific variables. These efforts would help optimize resource allocation, enhance precision in savings estimates, and ensure that retrofitting programs achieve their maximum potential in improving environmental sustainability and disaster resilience.

## 6 Conclusions

The Smart Hospitals Program effectively improved resource efficiency and sustainability in healthcare facilities through targeted retrofitting interventions, including rainwater harvesting, low-flow plumbing fixtures, LED lighting, inverter air-conditioning units, and photovoltaic systems. These measures significantly reduced water and energy consumption while enhancing resilience to disruptions caused by natural disasters or resource shortages.

Integrating infrastructure upgrades and staff training proved essential for maximizing long-term benefits. However, the need for continuous monitoring, robust maintenance, and tailored interventions was highlighted to sustain gains and address facility-specific challenges.

This program offers a replicable model for advancing sustainable healthcare infrastructure, emphasizing the importance of combining structural improvements with operational changes to reduce environmental impact and enhance system resilience.

## Ethics statement

The Smart Hospitals Program received ethical approval from each participating country's relevant national and institutional review boards. Written informed consent was obtained from facility managers for the inclusion of their data in the study.

## Funding

The project was funded by the UK Foreign, Commonwealth & Development Office (FCDO), which played a key role in supporting the implementation of the Smart Hospitals Program. Additionally, PAHO contributed significant personal time and effort and the Health Emergencies Department in Washington D.C., providing essential leadership and expertise.

## CRediT authorship contribution statement

**Judith Harvey:** Writing – original draft, Visualization, Validation, Resources, Methodology, Investigation, Formal analysis, Data curation, Conceptualization. **Dana van Alphen:** Writing – original draft, Validation, Software, Resources, Methodology, Investigation, Data curation, Conceptualization. **Shalini Jagnarine Azan:** Writing – original draft, Software, Resources, Methodology, Investigation, Formal analysis, Data curation. **Roger Camacho:** Writing – original draft, Software, Resources, Methodology, Investigation, Formal analysis, Data curation. **Clemens Buter:** Writing – original draft, Visualization, Software, Resources, Methodology, Investigation, Formal analysis, Data curation. **Ciro Ugarte:** Writing – original draft, Visualization, Resources, Project administration, Methodology, Investigation, Formal analysis. **Lealou Reballos:** Writing – original draft, Visualization, Software, Resources, Methodology, Investigation, Data curation. **Alex Camacho-Vasconez:** Writing – original draft, Validation, Supervision, Resources, Methodology, Investigation, Formal analysis, Data curation. **Juan S. Izquierdo-Condoy:** Writing – review & editing, Visualization, Validation, Supervision, Software, Investigation. **Jorge Vasconez-Gonzalez:** Writing – original draft, Visualization, Software, Resources, Methodology, Investigation. **Esteban Ortiz-Prado:** Writing – review & editing, Visualization, Validation, Supervision, Software, Resources, Methodology, Investigation.

## Declaration of competing interest

The authors declare that they have no known competing financial interests or personal relationships that could have appeared to influence the work reported in this paper.
